# Coinfection by *Mycobacterium tuberculosis* and *Rhodococcus equi* in advanced HIV

**DOI:** 10.1590/S1678-9946202668051

**Published:** 2026-08-03

**Authors:** Mariana Soares Kajita, Erika Yuki Mogami Bomfim, Gabriella Cecilia Vanin, João Vitor Matachon Viana, Guilherme Kenzo Shimizu Saito, Ceila Maria Sant’Ana Malaque, Jaques Sztajnbok

**Affiliations:** 1 Instituto de Infectologia Emílio Ribas, Unidade de Terapia Intensiva, São Paulo, São Paulo, Brazil

**Keywords:** HIV, Tuberculosis, Mycobacterium tuberculosis, Rhodococcus equi, Coinfection.

## Abstract

A 28-year-old man with HIV (diagnosed eight years prior, last CD4 count 23 cells/mm^3^) and prior tuberculosis (cured, then confirmedly reinfected with treatment default) presented with cachexia, diarrhea, chest pain, and tachypnea with increased work of breathing that required orotracheal intubation. Admission chest CT showed extensive bilateral consolidations with air bronchograms, suggestive of previous mycobacterial infection and/or possible new bacterial infection. Initially managed as sepsis, blood cultures grew *Rhodococcus equi* (four samples). Molecular testing confirmed *Mycobacterium tuberculosis* in sputum and tracheal aspirate, establishing coinfection and exceeding the similarities in acid-fast staining, clinical, and radiological findings between the two organisms. Treatment included antituberculosis therapy associated with clarithromycin and vancomycin (14 days). The patient achieved complete clinical improvement with partial radiological resolution (residual cavitations). He was discharged after 25 days, but was lost to follow-up and died three months later from probable septic shock. This report highlights a rare *M. tuberculosis*/*R. equi* coinfection in an immunosuppressed patient, emphasizing diagnostic challenges and the importance of therapeutic adherence in advanced HIV.

## INTRODUCTION

*Rhodococcus equi*, also referred to in some taxonomic literature as *Rhodococcus hoagii* ^1^ *,* is a gram-positive, partially acid-fast, obligate aerobic, catalase-positive, non-motile actinomycete found in soil and herbivore feces (particularly horses, deer, ruminants); it is also known as an opportunistic pathogen in a broad range of animal species^2^ ^),(^ ^3^. Infections in humans primarily affect immunocompromised patients, presenting as cavitary pneumonia (most common in HIV, transplant, and oncology patients), bacteremia (often catheter-associated), or disseminated disease^4^ ^),(^ ^5^. *R. equi* causes necrotizing cavitary pneumonia in 70%-75% of cases (50%-80% bacteremic cases in immunosuppressed patients), with reported mortality of 50%-61% in HIV patients, according to small case series (two-three patients each)^6^.

Risk factors include severe immunosuppression (CD4<200/mm^3^), diabetes mellitus, recent opportunistic infections, and animal exposure^3^. Diagnosis is challenging due to clinical and radiological features resembling other infections, morphological similarity to oropharyngeal diphtheroids (often dismissed as contamination), and partial acid-fastness, which may lead to misdiagnosis as a mycobacterial infection^6^ ^),(^ ^7^.

Treatment is based on the increasing resistance of strains and the intracellular nature of *R. equi*. Thus, it consists of two active drugs for a prolonged treatment duration, especially in immunocompromised patients (at least six months)^1^.

We report *M. tuberculosis*/*R. equi* coinfection in a 28-year-old man with AIDS (CD4 nadir 23/mm^3^), chemical dependency, and homelessness, highlighting diagnostic challenges in a tuberculosis (TB)-endemic setting.

### Ethics

Approval was obtained from the Institutional Research Ethics Committee of Emilio Ribas Institute of Infectious Diseases (process Nº 7.428.087), as was written informed consent from the legal representative for publication of the clinical description and images.

## CASE REPORT

A 28-year-old man with HIV (diagnosed eight years prior, irregularly treated with tenofovir disoproxil fumarate, lamivudine, and dolutegravir, and recurrent treatment defaults due to chemical dependency and homelessness, last CD4 count 23 cells/mm^3^ and viral load of 121 copies/mL from five months earlier, no genotyping performed), a history of pulmonary and lymph node tuberculosis (treated/cured eight years ago with a nine-month regimen; reinfection confirmed by sputum culture one year prior with treatment abandonment less than one month later; previous biomolecular testing showed no resistance to rifampin or isoniazid), and with reported domestic contact with chickens and cats presented to the emergency department with cachexia, liquid diarrhea, chest pain, and tachypnea with increased work of breathing (RR 58 breaths/minute with intercostal retractions). He reported the onset to be three months earlier with symptom worsening one week before admission. Orotracheal intubation was required within 24 h of admission due to respiratory failure.

Institutional sepsis protocol was initiated, with blood cultures (two pairs total: one pair of aerobic and one pair of anaerobic bottles), fluid resuscitation (1L normal saline), ceftriaxone 1g q12h + clarithromycin 500mg q12h (targeting a probable respiratory focus of infection), and restart of antituberculosis regimen (rifampin, isoniazid, pyrazinamide, ethambutol) due to patient’s history of treatment abandonment. Admission chest CT showed extensive bilateral consolidations with air bronchograms, thin bilateral pleural effusions, and minimal pericardial effusion, compatible with bacterial and mycobacterial infection in an immunocompromised host (Figure 1).

**Figure 1:**
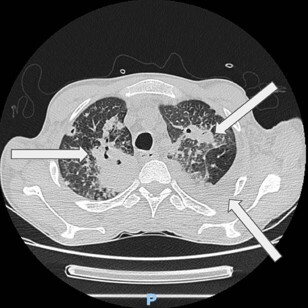
Chest computed tomography (CT) on admission showing extensive bilateral pulmonary consolidations with air bronchograms and thin pleural effusion.

Admitted to ICU for 17 days. Sputum (day one) and tracheal aspirate (day three) were positive for *M. tuberculosis* by Xpert^®^ MTB/RIF Ultra (AFB smear negative), maintaining susceptibility to rifampin. Blood cultures collected on days 1 (four samples), 3 (two samples), 11 (two samples), and 17 (two samples) were initially incubated in the BD BACTEC^™^ FX system. Results were positive within four days of incubation (one sample from day 1, one sample from day 3, and both samples from day 11 and day 17. All positive bottlers were aerobic). These positive samples were then subcultured into the BD Phoenix^™^ M50 system, which identified *R. equi*. Isolates from day 11 were also confirmed via MALDI-TOF. Susceptibility testing was unavailable. Empiric vancomycin replaced ceftriaxone for 14 days, with maintenance of clarithromycin to provide combined dual intravenous therapy against *R. equi* (immunocompromised patient). Antiretroviral therapy was restarted on day 12 with tenofovir disoproxil fumarate, lamivudine, and dolutegravir. Control CT (day 18) showed reduced consolidations with thick-walled irregular cavitations (largest 2.4×1.9cm left basal anterior), excavated nodules, reticulations, mild bronchiectasis (Figure 2).

**Figure 2:**
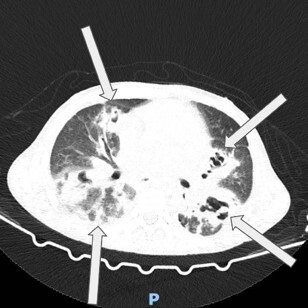
Chest CT on hospital day 18 showing partial resolution of consolidations with residual thick-walled cavitary lesions, excavated nodules, reticular opacities, and mild bronchiectasis.

Extubated on day nine, transferred to ward for eight days, discharged on day 25 for outpatient follow-up on azithromycin maintenance (500mg daily), antituberculosis treatment, and antiretroviral therapy targeting CD4>200/mm^3^. Total duration of treatment was expected to be six months, with monthly clinical consultations. Lost to follow-up; readmitted three months later with respiratory failure. The patient died on day four from presumably septic refractory shock.

## DISCUSSION

The literature review in this study aimed to address *R. equi* epidemiology, diagnostic challenges, and therapeutic approaches given the rarity of documented *M. tuberculosis*/*R. equi* coinfection. This 28-year-old man with advanced AIDS (CD4 nadir 23/mm^3^) presented with severe pneumonia, confirmed *M. tuberculosis/R. equi* coinfection by molecular testing and blood cultures, achieving initial clinical/radiological improvement with vancomycin + clarithromycin + antituberculosis therapy, succumbing to presumed septic shock after treatment default.

This case illustrates the critical importance of broad and diversified clinical reasoning when approaching patients living with HIV, particularly those in the AIDS phase with CD4 counts below 200 cells/mm^3^ due to the greater risk of coinfections (20%-30%) and overlapping clinical presentations among them^8^. Although *R. equi* pneumonia is mostly rapidly cavitary, it can also present without cavitation, resembling the pattern commonly seen in pulmonary tuberculosis among HIV-infected patients with CD4 lymphocytes <200 cells/μL (who also show a lower frequency of positive sputum smears)^9^. Our patient initially presented without cavitation, resembling pulmonary tuberculosis, but developed cavitary lesions within 20 days, a pattern more aligned with *R. equi* infection.

A retrospective analysis of 24 *R. equi* infections in HIV-positive patients found four key diagnostic challenges: (1) partial acid-fastness leading to erroneous mycobacterial diagnosis, (2) growth in sputum cultures dismissed as contaminants due to morphological similarity to diphtheroids, (3) early interstitial radiographic pattern suggesting alternative pneumonias, and (4) need for early empirical treatment delaying *R. equi* isolation^7^.

In Brazil, where resources are unevenly distributed (with limited access to advanced diagnostic tests) and tuberculosis is endemic, TB is often diagnosed on the basis of clinical findings, imaging, and acid-fast bacilli (AFB) positivity alone, especially in HIV-positive patients. In this case, the patient had a prior TB diagnosis and presented compatible clinical and radiological features. Even in the absence of AFB positivity, the clinical picture could have easily been misinterpreted as TB alone. Misdiagnosis was avoided only because a strict sepsis protocol ensured that blood cultures were collected before antibiotic initiation and because a specialized laboratory team distinguished true *R. equi* growth from contamination. Otherwise, suboptimal or incomplete response to antituberculosis therapy might have raised suspicion of either coinfection or resistance.

Although the vast majority of documented *R. equi* isolates remain susceptible to vancomycin, and over 90% remain susceptible to macrolides, imipenem, aminoglycosides, ciprofloxacin/levofloxacin, and rifampin, treatment of severe infections in immunocompromised or immunocompetent individuals with serious disease should include intravenous administration of two-three of these agents for the first weeks of a six-month or longer treatment^10^. In our case, vancomycin was added to the regimen in combination with clarithromycin as rifampin is only available in oral formulation. After discharge, azithromycin and rifampin were prescribed to complete treatment. 

Immune status likely represents the primary determinant of therapeutic success^11^ ^),(^ ^12^. In HIV/AIDS patients, the absence of adequate antiretroviral therapy has been associated with higher mortality, although disseminated *R. equi* disease has been documented even during potent antiretroviral therapy, when immunological recovery remains inadequate (CD4+ persistently <200/mm^3^)^13^. 

Coinfection with tuberculosis and *R. equi* remains rarely described and may be underdiagnosed in TB-endemic settings such as Brazil. In the sole prior report of coinfection, persistent AFB positivity despite antituberculosis treatment and clinical improvement prevented definitive conclusion regarding true coinfection versus pure *R. equi* infection with initially inadequate coverage^14^.

This case highlights the possibility of infection or coinfection by *R. equi*, which may present symptoms, radiological findings, and AFB positivity similar to those of tuberculosis, particularly among HIV/AIDS patients. Key lessons include: (1) rapidly developing cavitation, especially in severely immunocompromised patients, should raise suspicion of *R. equi* infection; (2) negative AFB does not exclude *R. equi* infection, and positive AFB does not confirm tuberculosis alone; (3) When available, Xpert^®^ MTB/RIF Ultra, TB-LAM, and blood cultures can be helpful in establishing a definitive diagnosis, particularly in TB-endemic countries^15^; and (4) therapeutic adherence and immunological recovery are likely key determinants of survival. 

## CONCLUSION

In conclusion, this single case report suggests the importance of broad differential diagnosis, early blood cultures, use of molecular tests such as Xpert^®^ MTB/RIF Ultra, and careful microbiological interpretation for accurate diagnosis and the role of antiretroviral therapy adherence and immunological recovery in treatment success. These inferences, however, are limited to a single patient, and further reports are needed to better define the clinical spectrum and management of *M. tuberculosis/R. equi* coinfection.

## Data Availability

The complete anonymized dataset supporting the findings of this study is included within the article itself.
